# Olfactory GPCRs through the lens of structural bioinformatics

**DOI:** 10.1093/chemse/bjaf038

**Published:** 2025-10-30

**Authors:** Alessandro Nicoli, Florian Bößl, Antonella Di Concilio Moschen, Francesco Ferri, Clarissa Rienaecker, Antonella Di Pizio

**Affiliations:** Section In Silico Biology & Machine Learning, Leibniz Institute for Food Systems Biology at the Technical University of Munich, Freising 85354, Germany; Professorship for Chemoinformatics and Protein Modelling, TUM School of Life Sciences, Technical University of Munich, Freising 85354, Germany; Section In Silico Biology & Machine Learning, Leibniz Institute for Food Systems Biology at the Technical University of Munich, Freising 85354, Germany; Professorship for Chemoinformatics and Protein Modelling, TUM School of Life Sciences, Technical University of Munich, Freising 85354, Germany; Section In Silico Biology & Machine Learning, Leibniz Institute for Food Systems Biology at the Technical University of Munich, Freising 85354, Germany; Professorship for Chemoinformatics and Protein Modelling, TUM School of Life Sciences, Technical University of Munich, Freising 85354, Germany; Section In Silico Biology & Machine Learning, Leibniz Institute for Food Systems Biology at the Technical University of Munich, Freising 85354, Germany; Professorship for Chemoinformatics and Protein Modelling, TUM School of Life Sciences, Technical University of Munich, Freising 85354, Germany; Section In Silico Biology & Machine Learning, Leibniz Institute for Food Systems Biology at the Technical University of Munich, Freising 85354, Germany; Professorship for Chemoinformatics and Protein Modelling, TUM School of Life Sciences, Technical University of Munich, Freising 85354, Germany; Section In Silico Biology & Machine Learning, Leibniz Institute for Food Systems Biology at the Technical University of Munich, Freising 85354, Germany; Professorship for Chemoinformatics and Protein Modelling, TUM School of Life Sciences, Technical University of Munich, Freising 85354, Germany

**Keywords:** odorant receptors, trace amine-associated receptors, deorphanization, AlphaFold, molecular docking, odorant recognition

## Abstract

Olfactory perception, mediated by G protein-coupled receptors (GPCRs) such as odorant receptors (ORs) and trace amine-associated receptors (TAARs), plays a pivotal role in human health, influencing behaviors like food choices and serving as early biomarkers for neurodegenerative diseases. Despite their importance, olfactory GPCRs are among the least understood members of the GPCR superfamily, and most ORs and TAARs are still orphan receptors. This review provides a comprehensive overview of recent advancements in the structural bioinformatics of olfactory GPCRs. We outline how computational, structure-based strategies have succeeded in identifying novel modulators for olfactory receptors. By discussing recent breakthroughs in GPCR structural biology, such as the first resolved experimental structures of ORs and TAARs, and the transformative impact of AI-driven structure prediction tools for olfactory receptors, this review offers a roadmap for future olfaction pharmacology research.

## 1. The GPCR branches behind our sense of smell: ORs and TAARs

Olfactory perception plays a critical role in human health and well-being, influencing behaviors such as food choices, memory recall, and emotional responses. The sense of smell enhances our experience of food and the environment, shaping dietary habits, and nutrition (Boesveldt and Parma 2021). Additionally, olfactory dysfunction can serve as an early indicator of neurodegenerative diseases such as Alzheimer's and Parkinson's (Dan et al. 2021; De Cleene et al. 2024). Beyond sensory perception, olfactory receptors are involved in various physiological processes in extranasal tissues, contributing to functions such as tissue regeneration and immune response (Massberg and Hatt 2018; Di Pizio et al. 2019). Therefore, understanding olfactory perception is vital for improving both health outcomes and quality of life.

**Fig. 1.**
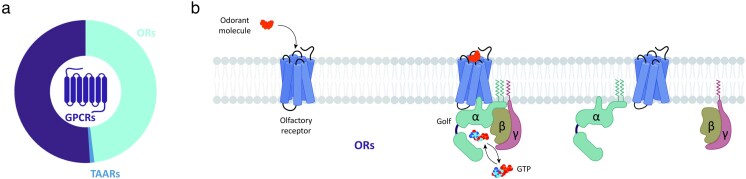
(a) Distribution of ORs and TAARs within the GPCR superfamily. (b) Schematic representation of the activation of olfactory GPCRs. Created in BioRender. Nicoli (2025) https://BioRender.com/ge24kix.

Odorant molecules are detected in the olfactory epithelium of mammals through repertoires of G protein-coupled receptors (GPCRs), i.e. the odorant receptors (ORs) and the trace amine-associated receptors (TAARs) (Buck and Axel 1991; Borowsky et al. 2001; Liberles and Buck 2006) (Fig. 1a). ORs were first unveiled in 1991 by the pioneering work of Richard Axel and Linda Buck, which was awarded the Nobel Prize in Medicine and Physiology in 2004 (Buck and Axel 1991). In 2006, Linda Buck also discovered members of the TAAR family in the olfactory epithelium (Liberles and Buck 2006). Since their discovery, many studies have followed aiming to investigate how these ORs and TAARs recognize odorants and decode the complex olfactory chemical system. As for other GPCRs, odorant molecules bind to the cognate receptor(s), and this leads to the recruitment of the G protein (Fig. 1b). The *G*_olf_ is the *G*_α_ subtype that couples with olfactory receptors in the olfactory epithelium, driving a cellular cascade of events (Jones and Reed 1989; Zhou et al. 2019). The information is then transduced from sensory cells into nerve signals to the brain (Francia and Lodovichi 2021). The olfactory system uses a combinatorial code of olfactory receptors to represent thousands of odorants: a specific receptor type may recognize more than one odorant, and each odorant may be recognized by more than one receptor (Malnic et al. 1999; Buck 2004; de March et al. 2020). Notably, each olfactory sensory neuron express only a single type of OR or TAAR (Pourmorady and Lomvardas 2022).

The exact number of functional ORs in humans is not defined as it depends on subpopulation specific expression patterns (Gilad and Lancet 2003). The NCBI Reference Sequence Database (https://www.ncbi.nlm.nih.gov/refseq/) contains 391 functional human OR sequences, and is the one we refer to in this manuscript. ORs can be phylogenetically categorized into two distinct groups whose amino acid sequence differences reflect their specific pattern of expression and odorant selectivity (Glusman et al. 2000; Fleischer et al. 2009; Olender et al. 2020). The first group, known as class I (so-called fish-like), comprises 56 receptors further divided into three subfamilies: 51, 52, and 56. The second group is the class II and contains all the remaining receptors, organized into 14 subfamilies, denoted as OR1-14 (Cichy et al. 2019; Niimura and Nei 2003).

Twenty-six subtypes of TAARs have been identified in mammalian species and categorized into nine different subfamilies (TAAR1-9) (Borowsky et al. 2001; Lindemann et al. 2005; Liberles and Buck 2006). The number of expressed TAARs varies among different species; humans only exhibit six functional isoforms, with TAAR3, 4 and 7 subtypes appearing to be pseudogenes (Lindemann et al. 2005). The TAAR family is thought to derive from the closest monoaminergic homolog in vertebrates, the serotonergic receptor 4 (5-HT_4_) (Hashiguchi and Nishida 2007; Dieris et al. 2021). The name of this class of receptors originates from TAAR1, the first deorphanized receptor in 2001, which was found to respond to the biogenic trace amines (TAs) (Borowsky et al. 2001; Bunzow et al. 2001). The term “trace amines” was initially coined to refer to endogenous amines with tissue levels below 100 ng/g, lower concentrations (<10 ng/tissue) than canonical biogenic amines, like dopamine, epinephrine, norepinephrine, and serotonin in the brain. TAs are also present in decaying foods and animal body fluids due to the decarboxylation of amino acids by endogenous enzymes or microbial metabolism (Liberles 2015).

TAAR1 is the only TAAR member not expressed in the nose and is widespread in mammalian brain (Borowsky et al. 2001; Gainetdinov et al. 2018). TAAR1 is a recognized drug target for mental disorders (including schizophrenia, depression, bipolar disorder and drug addiction) (Gainetdinov et al. 2018; Nair et al. 2022; Liu et al. 2024). TAAR2-9 members have been initially detected in murine olfactory sensory neurons and are therefore defined as noncanonical olfactory receptors. TAAR2-9 play an essential function in detecting volatile amines linked to ethological or ecological signals (Ferrero et al. 2011; Dewan et al. 2013; Li et al. 2013; Dewan 2021).

Recent studies have revealed the presence of olfactory TAARs (TAAR2-9) in diverse extranasal tissues, where they are suggested to be involved in inflammatory responses initiated by dietary factors (e.g. Crohn's disease and ulcerative colitis), metabolic disorders (e.g. type-2 diabetes and obesity), immune system related diseases, and even melanoma and breast cancer (Berry et al. 2017; Gainetdinov et al. 2018). ORs are also increasingly recognized for their expression in extranasal tissues. These receptors have been identified in various organs, including the heart, liver, gut, and skin, where they contribute to a range of physiological functions, such as tissue regeneration and immune response, beyond olfactory perception (Di Pizio et al. 2019; Lee et al. 2019).

Despite their broad distribution and potential relevance in health and disease, olfactory GPCRs remain among the most understudied members of the GPCR superfamily (Scharf et al. 2025). Recent advancements in GPCR structure biology and advances in techniques such as molecular modeling, molecular dynamics (MD) simulations, and AI-driven structure prediction offer new opportunities to unravel the complexities of their function (Kogut-Gunthel et al. 2025). In this review, we provide a comprehensive overview of the current state of olfactory receptor computational research, highlighting key discoveries, advancements, and future perspectives.

## 2. Sequence landscape of olfactory GPCRs

GPCRs constitute the largest family of membrane proteins in the human genome, with over 800 members, and are characterized by a conserved architecture of seven transmembrane (TM) α-helices connected by three intracellular loops and three extracellular loops (ECLs). Among the six phylogenetically defined GPCR classes—A (rhodopsin-like), B (secretin-like), C (metabotropic glutamate receptors), D (pheromone receptors), E (cAMP receptors), and F (frizzled/smoothened receptors) (Fredriksson et al. 2003).

Class A is the most numerous, comprising over 719 members (Yang et al. 2021). Class A GPCRs exhibit conserved TM sequence motifs, known as molecular switches, that underlie shared structural features and activation mechanisms (Zhou et al. 2019). The Ballesteros–Weinstein numbering scheme is commonly used for this class to standardize residue positions, assigning the most conserved residue in each TM helix the index X.50, where X corresponds to the helix number. GPCRs demonstrate remarkable diversity in structure, function, and ligand specificity, ranging from single protons to large proteins. The most conserved residues are N^1.50^, D^2.50^, R^3.50^, W^4.50^, P^5.50^, P^6.50^, and P^7.50^, playing essential roles in receptor structure and function (Isberg et al. 2015). Long-range intramolecular and intermolecular interactions at distant sites on the same receptor are crucial factors that modulate signaling function of GPCRs. Positive or negative coupling between the extracellular, the transmembrane and the intracellular domains facilitates cooperativity of activating “switches” as requirements for the functional plasticity of GPCRs (Unal and Karnik 2012).

**Fig. 2.**
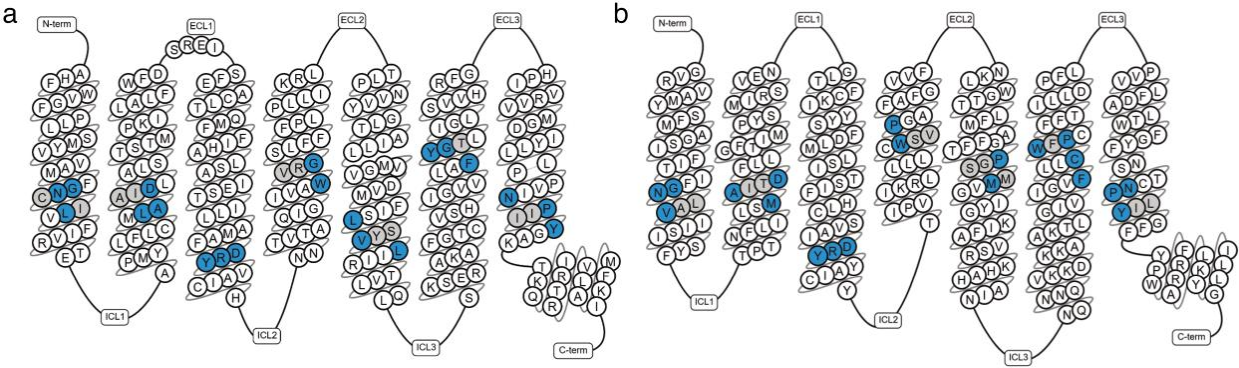
Macroswitches are highlighted in the snake plots of (a) OR (OR51E2 sequence) and (b) TAAR (TAAR2 sequence). The plots were generated using the “snake plot” tool in GPCRdb (https://gpcrdb.org/protein/taar2_human/).

Olfactory GPCRs are the most numerous class A GPCRs, accounting for more than half of the entire GPCRome (Fig. 1a). ORs share <20% sequence identity with nonolfactory class A GPCRs. In contrast, TAARs share ∼25% to 30% sequence identity with class A GPCRs (de March et al. 2015; Eyun et al. 2016; Scharf et al. 2025). A comparison of macroswitches in ORs and TAARs compared to other class A GPCRs is reported in Table 1 and Fig. 2.

**Table 1.** Macroswitches in olfactory GPCRs.

	*Class A GPCRs*	*ORs*	*TAARs*
**TM1**	GNxxV	GNxxL	GNxxV
**TM2**	LAxxD	LSxxD	LAxxD
**TM3**	DRY	DRY	DRY
**TM4**	Wxxx	WxxG	WxxP
**TM5**	PxxxI (*PIF motif*)	LxxVL	PxxxM (*PIF motif*)
**TM6**	F(*PIF motif*), CWxP	FYGx	F(*PIF motif*), CWxP
**TM7**	NPxxY	NPxIY	NPxxY

GPCR macroswitches are all conserved in TAARs (Table 1 and Fig. 2), despite some intrafamily differences (e.g. the L^2.46^xxxD^2.50^ is M^2.46^xxxD^2.50^ in TAAR1 and TAAR2, the D^3.49^R^3.50^Y^3.51^ motif is replaced by the DRF and DRH motifs in TAAR2 and TAAR5, respectively), which are anyway reported in other GPCRs.

Most of the conserved residues in class A GPCRs are also present in ORs (Table 1). Noteworthy, TM6 is the most divergent helix when compared to other GPCRs, whereas it is highly conserved within OR family: the conserved F^6.44^xxC^6.47^W^6.48^xP^6.50^ motif is replaced by the V^6.44^xxF^6.47^Y^6.48^G^6.49^×^6.50^ motif in ORs.

Importantly, the PIF motif (I^3.40^, P^5.50^, and F^6.44^) involved in the activation mechanism of class A GPCRs is not present in ORs (Wacker et al. 2013; Billesbolle et al. 2023). In class I ORs, these residues are replaced by the highly conserved residues S^3.40^, D^5.50^, V^6.44^, which are instead variable positions in class II ORs. This motif is also different for different TAAR members, and it is PIY, PLF, PVF, and PLF for TAAR5, TAAR6, TAAR8, and TAAR9, respectively.

## 3. Molecular receptive range of olfactory GPCRs

The molecular receptive range is the set of chemical compounds (odorants) that bind to and stimulate a specific olfactory receptor. With their extensive repertoire of olfactory GPCRs, humans can distinguish numerous odorant molecules, estimated to be 10,000, or even over one trillion (Bushdid et al. 2014; Crocker and Henderson 2002). This remarkable capability is suggested to be achieved through the combinatorial code of odorant recognition; each olfactory GPCR can bind multiple odorants, and each odorant can interact with multiple receptors (Malnic et al. 1999; de March et al. 2020). The advancements in cell-based and cell-free assays, instruments and techniques are leading to a constant growth of ligand knowledge (Di Pizio et al. 2020). However, most olfactory GPCRs are still orphan (Lalis et al. 2024a; Scharf et al. 2025).

TAAR1 is a recognized drug target and therefore many ligands were identified in the attempt to design a TAAR1-targeted drug (Diaz-Holguin et al. 2024; Scarano et al. 2024). Its agonist ralmitaront (RO6889450) is currently in phase 2/3 trials for the treatment of schizophrenia (Achtyes et al. 2023; Halff et al. 2023). Olfactory TAARs (2-9) play an essential function in detecting volatile amines linked to ethological or ecological signals (Dewan et al. 2013; Li et al. 2013). Due to their functional significance, murine TAARs have been more extensively characterized in terms of their molecular receptive range compared to human TAARs (Xu and Li 2020; Guo et al. 2022). However, the discovery of olfactory TAAR2-9 receptors in various extranasal tissues, where they are involved in key physiological and pathological processes, highlights the urgent need for deorphanization efforts targeting these receptors (Alnefeesi et al. 2024).

Similarly, only a small fraction of ORs have been deorphanized so far, and the combinatorial code is known for very few odorants. M2OR (https://m2or.chemsensim.fr/), a database with a curated collection of OR ligands sourced from published literature (51,483 unique pairs across 11 mammalian species and receptor variants) has been recently released (Lalis et al. 2024b). The analysis of ligand information of human ORs demonstrates that so far only 12% of human ORs have been deorphanized via experimental screening, and 4.5% via dose-response assays (Lalis et al. 2024a). Some OR families (e.g. families 1, 9, 21, and 56) are better studied than others (e.g. families 3 and 12). Most reported EC_50_ values are in the micromolar to millimolar range, and it is difficult to line a clear correlation between EC_50_ and odorant olfactory descriptors (e.g. floral and woody) (Lalis et al. 2024a).

Among the deorphanized ORs, we can identify broadly tuned ORs that were found to bind many ligands and narrow ORs that are highly selective against specific odorant molecules. With 153 reported agonists, OR2W1 is the most broadly tuned human OR known (Haag et al. 2022). Its agonists are chemically diverse but represent an exclusive receptive range, complementary to chemical subgroups covered by evolutionary younger, highly selective receptors. The analysis of all human OR ligands in the M2OR database suggests that food-derived odorants often activate few ORs, while floral odorants activate many ORs, aiding in environmental sensing. These analyses together suggests that broadly tuned ORs could play a crucial role in the perception of diverse odor categories, including food-related key food odorants (KFOs), off-flavors, body odors, semiochemicals, ambient odors, and warning signals for danger and poison. Since these odors are typically experienced as complex mixtures, both specialist and generalist ORs are required for effective combinatorial odorant coding at the receptor level. This combinatorial mechanism allows the brain to recognize odor-specific patterns, enabling the discrimination of both individual odorants and complex odor bouquets.

## 4. New olfactory GPCR modulators identified via structure-based virtual screening

Computational structure-based approaches, such as molecular docking, MDs simulations, and virtual screening, have markedly advanced our knowledge of GPCR function, activation, and modulation (Ballante et al. 2021; Kogut-Gunthel et al. 2025; Lopez-Balastegui et al. 2025). However, progress in the olfactory GPCR field has lagged behind. This is largely attributable to the scarcity of known ligands but also the absence of high-resolution receptor structures (Scharf et al. 2025). To date, most computational studies of ligand interactions with ORs and TAARs have relied on homology models derived from more closely studied class A GPCR templates, such as adrenergic receptors (Scarano et al. 2024). Yet, as discussed above, olfactory GPCRs are rather different from other class A GPCRs, posing substantial challenges for accurate structural modeling. Despite this challenge, computer-aided screenings have enabled the successful identification of novel modulators for olfactory receptors.

A virtual screening campaign of >2,500 metabolites against the structure model of OR51E2 identified 24 novel agonists and 1 antagonist for this receptor (Abaffy et al. 2018). The β₂-adrenergic receptor (4LDO) was used as the template for homology modeling, two scoring functions (Score and mfScore) were combined to enhance ligand prediction accuracy in the docking screening, and the top 50 hits from each scoring list were selected for experimental validation. 19-Hydroxyandrostenedione (19-OH AD) (a testosterone metabolite) and N-acetyl-N-formyl-5-methoxykynurenamine (AFMK) (a melatonin metabolite) were found among the newly identified agonists (Abaffy et al. 2018). OR51E2 is highly expressed in prostate cancer, including castration-resistant and neuroendocrine prostate cancer. The identification of endogenous metabolites that modulate OR51E2 reveals a novel role for this receptor in driving neuroendocrine differentiation in prostate cancer, highlighting its potential as a therapeutic target for aggressive prostate cancer subtypes.

OR51E2 was also the target of a recent virtual screening campaign that led to the identification of the first intracellular modulator of an OR (Abaffy et al. 2024). The virtual screening used an AI protocol based on deep convolutional neural network from the AtomNet screening platform (Atomwise®) against a ∼2.2 million small-molecule library (enamine_instock_v200204). The identified compound acts as a negative allosteric modulator (NAM) and binds to an allosteric binding site formed by a network of nine residues localized in the intracellular parts of transmembrane domains 3, 5, 6, 7, and H8, which also partially overlaps with a G protein binding site (Abaffy et al. 2024).

Cong et al. (2022) have suggested a supervised machine learning (ML) model based on OR sequence similarities and ligand physicochemical features to deorphanize ORs. The study aimed to decode how olfactory receptors recognize diverse odorants by leveraging ML and proteochemometric (PCM) modeling. The model was based on 1293 OR-odorant pairs (390 ORs, 244 odorants) from literature, including 14,400 nonresponsive pairs. The binding site residues (17-60) were identified using homology models of mOR256-31 receptors and mutagenesis data. Trained Random Forest (RF) and Support Vector Machine classifiers using PCM were built combining OR sequence features and odorant physicochemical properties. Experimental validation confirmed micromolar-to-millimolar EC_50_ values for new pairs, demonstrating that this approach can perform well for new ORs but requires more training data for new odorants.

Computer-guided receptor-based screenings were also attempted for the deorphanization of olfactory TAARs. Especially, virtual screening campaigns have been focused on TAAR5. TAAR5's expression in major limbic brain areas links it to the regulation of emotional behavior, suggesting potential therapeutic applications for anxiety and depression (Espinoza et al. 2020; Maggi et al. 2022). Furthermore, there is evidence suggesting a correlation between mTAAR5 (mouse TAAR5), adult neurogenesis, dopamine transmission, and sensorimotor and cognitive functions (Efimova et al. 2021; Kalinina et al. 2021). The discovery of additional ligands is crucial for a comprehensive understanding of TAAR5 function. Currently, few ligands (agonists and antagonists) are known for the mouse TAAR5 (Gainetdinov et al. 2018; Xu and Li 2020). Given the high sequence identity between mTAAR5 and hTAAR5 (human TAAR5), insights gained from mTAAR5 can be applied to the human counterpart (Nicoli et al. 2023b).

Cichero and coworkers conducted the first structure-based virtual screening targeting murine TAAR5, using a 3D model built via comparative modeling with the structure of β_2_-adrenoreceptor (β_2_-AR) as a template (Cichero et al. 2016). An in-house library of five-membered ring derivatives linked to a phenyl ring was screened using Surflex docking in Sybyl-X 1.0. The top-ranked compounds were tested in HEK-293 cells expressing mTAAR5 or mTAAR1, leading to the discovery of the first two mTAAR5 antagonists with low micromolar activity.

A virtual screening campaign targeting mTAAR5 was more recently performed by applying the AtomNet® screening platform (Bon et al. 2022). The structural model of mTAAR5 was built using *Meleagris gallopavo* β_1_-AR as the template. Top 94 candidates were tested via BRET assay (cAMP inhibition) and the downstream signaling (ERK/CREB phosphorylation) was assessed, leading to the identification of two novel TAAR5 antagonists with low micromolar potency.

Recently, a focused virtual screening campaign disclosed three novel mTAAR5 antagonists based on diverse chemical scaffolds (Nicoli et al. 2023b). The structure of mTAAR5 was predicted using multitemplate homology modelling with β_1_-AR and β_2_-AR as templates. The starting models were refined with induced-fit docking simulations with known ligands to improve the conformational sampling of the binding site. The refinement work was driven by insights derived from comparing TAAR5 and serotonin receptors, as the mTAAR5 ligand also acts as a modulator of serotonin receptors. Combining sequence- and structure-based analyses, two sets of six and eleven residues to be sampled with the induced-fit docking simulations were identified, 1,491 models were generated and assessed, and the best-performing models (A and B) were selected for the virtual screening campaign. The virtual screening campaign consisted of filtering steps that brought 18,969 compounds to the docking. Twenty-nine compounds were experimentally tested, and three new mTAAR5 antagonists were discovered with a hit rate of 10%. The binding modes of the new antagonists were characterized through postdocking MD simulations. The newly identified ligands can pose the basis for the development of TAAR5 drug design and for studying the physiological roles of TAAR5.

## 5. Structural and conformational landscape of olfactory GPCRs

As described above, structure-based ligand design studies have relied on OR model structures until now. However, recent advancements in GPCR structure biology have led to the publication of the first OR structures (Table 2). These structures will pave the way for new sources of information in modeling investigations.

**Table 2.** List of all published odorant receptor experimental structures. Most solved structures are consensus OR (consOR) structures.

	PDB ID	Ligand	Resolution (Å)	G protein	Ref.
**OR51E2**	8F76	propionate	3.10	miniG_s399_	Billesbolle et al. (2023)
**consOR51**	8UXV	(apo)	3.20	miniG_s399_	de March et al. (2024)
**consOR1**	8UXY	L-menthol	3.30	miniG_s399_	de March et al. (2024)
**consOR2**	8UY0	S-carvone	3.20	miniG_s399_	de March et al. (2024)
**consOR4**	8UYQ	2-Methylthiazoline	3.50	miniG_s399_	de March et al. (2024)
**consOR52**	8HTI	Octanoic acid	2.97	G_s_	Choi et al. (2023))
**consOR52**	8J46	(apo)	3.66	–	Choi et al. (2023)
**consOR52**	8W77	(apo)	3.61	–	Choi et al. (2023)

Currently, most of the available OR structures (4 out of 5 unique ORs) were obtained for consensus sequences (i.e. sequences representing the most conserved residues across OR subfamilies) (Table 2). The determination of consensus ORs of class I (i.e. consOR51 and consOR52) and class II (i.e. consOR1, consOR2, and consOR4) enabled the comparison between OR subfamilies for the first time, while the structures in different conformational states e.g. consOR51 in active and inactive state give insights into their activation mechanism (Choi et al. 2023; de March et al. 2024).

The advancements in GPCR structural biology also led to the characterization and determination of the first structures of TAARs in 2023 (Table 3).

**Table 3.** List of all published TAAR structures.

	PDB ID	Ligand	Resolution (Å)	G protein	Refs.
**hTAAR1**	8JLN	T1AM^1^	3.24	G_s_	Xu et al. (2023)
**hTAAR1**	8JLO	ulotaront	3.52	G_s_	Xu et al. (2023)
**hTAAR1**	8JLP	ralmitaront	3.23	G_s_	Xu et al. (2023)
**hTAAR1**	8JLQ	fenoldopam	2.84	G_s_	Xu et al. (2023)
**hTAAR1**	8JLR	A77636	3.00	G_s_	Xu et al. (2023)
**hTAAR1**	8JSO	(S)-AMPH^2^	3.40	G_s_	Xu et al. (2023)
**hTAAR1**	8UHB	RO-5256390	3.35	G_s_	Zilberg et al. (2024)
**hTAAR1**	8W87	METH^3^	2.80	G_s_	Liu et al. (2023)
**hTAAR1**	8W88	ulotaront	2.60	G_s_	Liu et al. (2023)
**hTAAR1**	8W89	PEA^4^	3.00	G_s_	Liu et al. (2023)
**hTAAR1**	8W8A	RO-5256390	2.80	G_s_	Liu et al. (2023)
**hTAAR1**	8WCA	PEA^4^	3.48	G_s_	Shang et al. (2023)
**hTAAR1**	8WC8	ZH8651	2.90	G_s_	Shang et al. (2023)
**hTAAR1**	8ZSJ	(apo)	2.80	Gs	Jiang et al. (2024)
**hTAAR1**	8ZSP	LSD^5^	3.14	G_s_	Jiang et al. (2024)
**hTAAR1**	8ZSS	RO5263397	3.07	G_s_	Jiang et al. (2024)
**hTAAR1**	9JKQ	METH^3^	2.66	G_s_	Lin et al. (2024)
**mTAAR1**	8JLJ	T1AM^1^	3.10	G_s_	Xu et al. (2023)
**mTAAR1**	8JLK	ulotaront	3.22	G_s_	Xu et al. (2023)
**mTAAR1**	8WC3	ulotaront	3.00	G_s_	Shang et al. (2023)
**mTAAR1**	8WC4	ZH8651	3.10	G_s_	Shang et al. (2023)
**mTAAR1**	8WC5	TMA^6^	3.30	G_s_	Shang et al. (2023)
**mTAAR1**	8WC6	PEA^4^	3.20	G_s_	Shang et al. (2023)
**mTAAR1**	8WC7	ZH8667	3.10	G_s_	Shang et al. (2023)
**mTAAR1**	8WC9	ZH8651	3.20	G_q_	Shang et al. (2023)
**mTAAR1**	8WCB	CHA^7^	3.10	G_q_	Shang et al. (2023)
**mTAAR1**	8WCC	CHA^7^	3.04	—	Shang et al. (2023)
**mTAAR1**	8ZSV	RO5263397	2.96	G_s_	Jiang et al. (2024)
**mTAAR7f**	8PM2	DMCH^8^	2.92	G_s_	Gusach et al. (2024)
**mTAAR9**	8ITF	DMCHA^8^	3.46	G_s_	Guo et al. (2023)
**mTAAR9**	8IW1	PEA^4^	3.40	G_olf_	Guo et al. (2023)
**mTAAR9**	8IW4	SPE^9^	3.49	G_s_	Guo et al. (2023)
**mTAAR9**	8IW7	PEA^4^	2.97	G_s_	Guo et al. (2023)
**mTAAR9**	8IW9	CAD^10^	3.08	G_s_	Guo et al. (2023)
**mTAAR9**	8IWE	SPE^9^	3.40	—	Guo et al. (2023)
**mTAAR9**	8IWM	PEA^4^	3.17	G_s_	Guo et al. (2023)

1: 3-iodothyronamine; 2: (S)-amphetamine; 3: methamphetamine; 4: β-phenylethylamine; 5: lysergic acid diethylamide; 6: tetramethylammonium; 7: cyclohexylamine, 8: N,N-dimethylcyclohexylamine; 9: spermidine; 10: cadaverine.

**Fig. 3.**
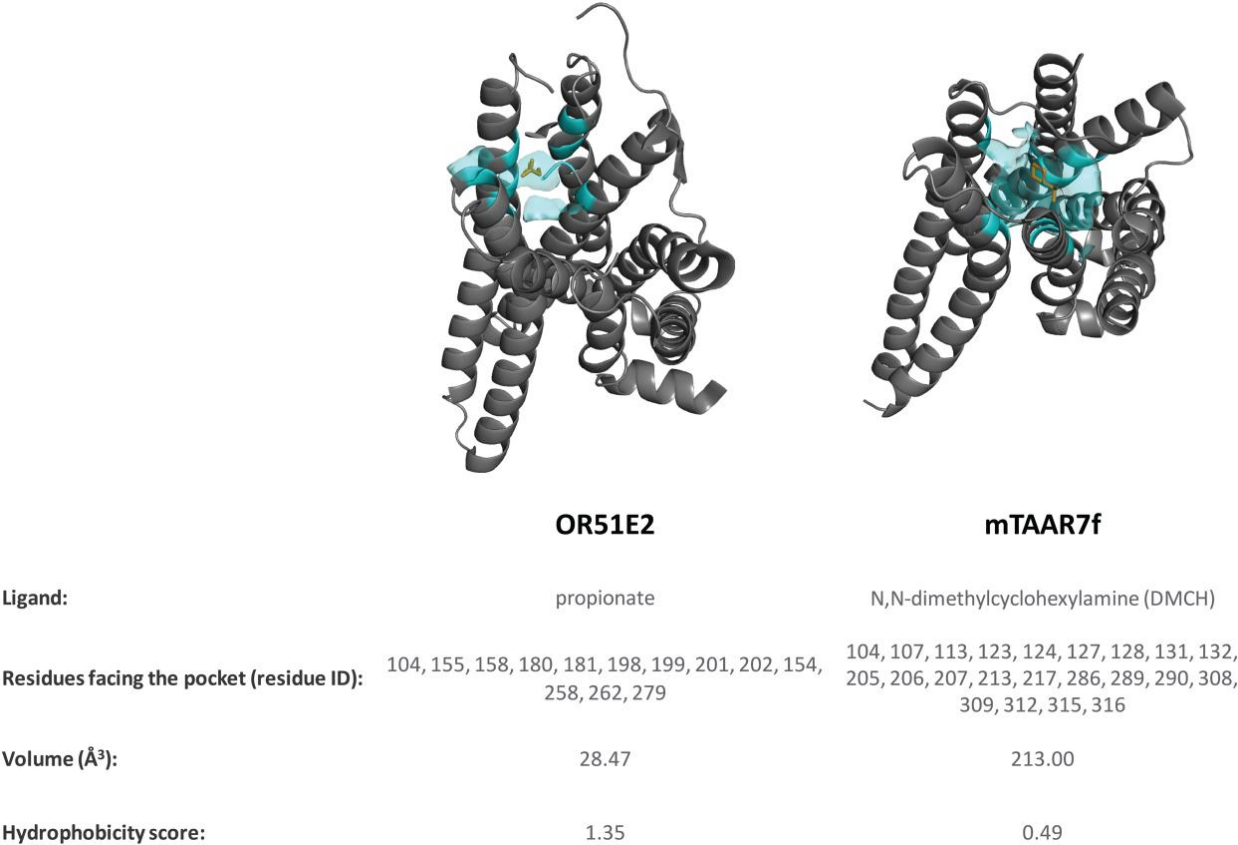
3D structural representation of OR51E2 (PDB ID: 8F76) and mTAAR7f (PDB ID: 8PM2) with highlighted the ligand binding pocket. Propionate (OR51E2 ligand) and DMCH (mTAAR7f ligand) are shown in orange stick. The ligand binding site (residues surrounding the ligands within a 4 Å radius) is shown in cyan transparent surface. The pocket descriptors were computed with SiteMap (Halgren 2009) (Schrödinger Release 2025-1: SiteMap, Schrödinger, LLC, New York, NY, 2025).

Different from ORs, TAAR structures revealed a canonical ligand binding site more similar to the orthostheric binding site of class A GPCRs (Fig. 3).

The availability of experimental structures has enabled the evaluation of the performance of computational modeling approaches to olfactory receptors. Interestingly, the binding site residues identified in the modeling work by Cong et al. (2022) overlap with the orthosteric binding site identified by the experimental structures (Fig. 3). Similarly, the hydrophobic patch formed by the residues F278^6.51^, F287^7.34^, and I291^7.38^ in the mTAAR5 binding site (Nicoli et al. 2023b) aligns to the coordinates of these residues in the mTAAR9 (root mean square deviation of sidechains atoms: 1.19). This suggests that extensive sampling and experimental integration in the modeling succeeded to model ligand-receptor interactions. However, variable and unstructured domains, such as the ECL2 (Nicoli et al. 2022), could not be properly modelled, hampering the use of advanced MD simulations to these homology models.

**Fig. 4.**
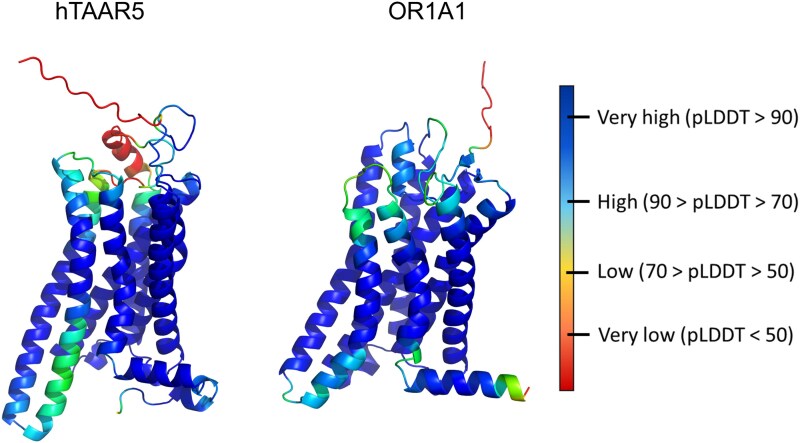
AlphaFold2 structure prediction for a TAAR5 (left) and an OR (right) structure colored by confidence scores. Human TAAR5 and OR1A1 are selected as examples. AlphaFold uses pLDDT (per-residue measure of local confidence) values as measures of confidence: it is scaled from 0 to 100, with higher scores indicating higher confidence and usually a more accurate prediction.

Currently, only a small fraction of the vast olfactory receptor family is covered by experimental structures. Artificial intelligence (AI)-based methods are proving to be powerful resources for predicting the three-dimensional architecture of proteins (Hameduh et al. 2020; Baek et al. 2021). This was underlined by the recent award of the Nobel Prize in Chemistry 2024 to Demis Hassabis and John M. Jumper for the development of AlphaFold 2 (AF2), which has demonstrated the ability to predict protein domain structures with accuracy comparable to experimental methods (Jumper et al. 2021). By providing accurate structural models for virtually all members of the OR family, AlphaFold helps to fill critical gaps in our structural knowledge and facilitates hypothesis-driven research across olfaction-related fields (Kogut-Gunthel et al. 2025). As a coevolution-based approach, the AF2 models already provide insights in regions that are more selective within the OR and TAAR subfamilies of olfactory receptors. In Fig. 4, we report the AF2 models of TAAR5 and OR1A1. By comparing the structures, the difference in structure prediction confidence of different domains becomes apparent: while ECLs are predicted with high confidence in OR1A1, the extracellular domains of hTAAR5 are predicted with medium to low confidence.

In a recent paper from our group, the applicability of traditional vs. AI structural modeling for structure-based studies of ORs was evaluated using OR5K1 as a case study (Nicoli et al. 2023a). A well-characterized dataset of chemically similar agonists and inactive compounds for OR5K1 were made available through a HTS campaign (Marcinek et al. 2021). We combined ligand information and mutagenesis data to guide an extensive conformational sampling (over three rounds of induced-fit docking) of the binding site of OR5K1 starting from a model structure obtained from homology modeling and a model structure obtained from AF2. This work provided not only an interesting comparison of protein structure prediction methods for ORs, but also a protocol for model optimization that was found to be necessary in both cases. Two conserved leucine residues, L104^3.32^ and L255^6.51^, which are conserved in 98% and 96% of OR5K1 orthologs across 51 species, respectively were experimentally mutated to alanine, confirming their involvement in the predicted binding modes.

AF2 models were also used to elucidate the binding modes of the two enantiomers of the KFO sotolone within OR8D1 (Wang et al. 2024). Functional assays revealed that the S-enantiomer has higher potency than the R-enantiomer against OR8D1 (EC_50_ values of 84.98 ± 1.05 and 167.20 ± 0.25 µmol/L, respectively). Binding site location and ligand binding modes were predicted by sequence analysis and molecular docking, followed by postdocking MD simulations. Ligand stability within the binding pocket was further assessed using Molecular Mechanics/Poisson–Boltzmann Surface Area (MM-PBSA) analysis. Key binding residues identified computationally were mutated in in vitro studies revealing key position in TM3, TM4, TM5, and TM6 for ligand recognition and enantioselectivity of OR8D1 toward sotolone.

Like all GPCRs, olfactory receptors undergo dynamic transitions between multiple conformational states, which are crucial for ligand binding, activation, and signaling (Zhou et al. 2019; de March et al. 2024). Understanding this conformational landscape is essential for accurate modeling of OR function and for rational ligand design. AF2 structures were successfully used also to simulate the inactive state of the OR OR51E2 (Alfonso-Prieto and Capelli 2023; Pirona et al. 2024). Structural models from AlphaFold, RoseTTAFold, OmegaFold, ESMFold, and AlphaFold-MultiState and an homology model from SwissModel were simulated for 500 ns with and without sodium ions in the conserved sodium binding pocket in class A GPCRs. Models without sodium showed structural collapse (TM6-TM7 interface disruption) due to water influx, while sodium-bound models maintained stability, confirming the ion's role in preserving the inactive conformation. This work highlights the relevance of receptor dynamics and proved the improved accuracy of state-specific training.

Advancements in AI-driven modeling continue to progress (Sim et al. 2025). For an updated list of the latest AI-based tools for molecular modeling applications, please refer to our repository: https://github.com/DiPizio-Lab/List_AI_Tools. There have been many advancements in AI-based ligand binding prediction, including version 3 of AlphaFold (AF3) (Abramson et al. 2024), RoseTTAFold-all-atom (Krishna et al. 2024), and Boltz-2 (Wohlwend et al. 2024; Passaro et al. 2025). Integrating these AI-based tools with the ever-expanding chemical space (Gentile et al. 2020; Luttens et al. 2025) holds great promise for virtual screening pipelines targeting olfactory receptors. This can speed up the process of identifying novel ligands and improve our understanding of orphan receptor functions and structures. Furthermore, integrating static structures from experimental or modeling sources with MD simulations and other techniques that capture protein flexibility is essential to comprehensively understanding olfactory GPCR behavior.

## 6. Conclusions and perspectives

Despite significant progress in recent years, ∼88% of ORs and most of TAARs remain orphans, lacking identified ligands. Breakthroughs in structural biology and AI-driven protein structure prediction, such as AlphaFold, have dramatically advanced our ability to model OR structures. This progress represents a turning point in the field of olfaction, as structural information enables deeper insight into the molecular recognition mechanisms of odorant molecules and the broader molecular coding of smell. Previous structural investigations suggested that applying conformational sampling and refinement protocols to predicted olfactory GPCR structures can lead to improved model performance in virtual screening and ligand discovery. These results support the hypothesis that dynamic modeling of receptor conformations enhances the predictive power of computational deorphanization efforts. As AI-based tools and experimental data continue to evolve, their integration has the potential to significantly accelerate and reduce the cost of ligand identification for orphan olfactory GPCRs. Ultimately, this approach brings us closer to resolving long-standing questions around olfactory receptor activation and odor coding at the molecular level.

## Data Availability

No new data were generated or analyzed in support of this research.
